# A Rare Case of A Low-Grade Inflammatory Leiomyosarcoma/Histiocyte-Rich Rhabdomyoblastic Tumor in the Neck of An Adolescent Male

**DOI:** 10.5146/tjpath.2022.01577

**Published:** 2023-05-15

**Authors:** Bharat Rekhi, Munita Bal, Bhaskar Dharavath, Amit Dutt, Prathamesh Pai

**Affiliations:** Department of Pathology Tata Memorial Hospital, Mumbai, India; Homi Bhabha National Institute (HBNI) University, Mumbai, India; Homi Bhabha National Institute (HBNI) University, Mumbai, India; Integrated Genomics Laboratory, Advanced Centre for Treatment, Research and Education In Cancer, Tata Memorial Centre, Navi Mumbai, India; Department of Surgical Oncology (Head and Neck Disease Management Group), Tata Memorial Hospital, HBNI University, Mumbai, India

**Keywords:** Soft tissue neoplasms, Neck, Leiomyosarcoma, Rhabdomyosarcoma, Histiocytes

## Abstract

Inflammatory leiomyosarcoma (LMS) is a newly included rare tumor entity in the group of smooth muscle tumors in the recent WHO classification. Recent studies have shown skeletal muscle expression within this tumor and its proximity with histiocyte-rich rhabdomyoblastic tumor (HRRT).

A 17-year-old male presented with a soft tissue lump over the back of his neck of one-year duration. Radiologically, a lesion measuring 5.9 cm in the largest dimension was seen, extending from the skull base up to the C2 vertebral level, abutting the occipital bone. The initial biopsy was reported as a fibrohistiocytic tumor at the referring laboratory. A microscopic review of the sections from the initial biopsy and subsequent resection revealed a well-circumscribed, cellular tumor composed of plump spindle and polygonal-shaped tumor cells with relatively bland nuclei, moderate to abundant eosinophilic cytoplasm and numerous interspersed histiocytes, including foam cells and lymphocytes. Immunohistochemically, the tumor cells were positive for desmin, MYOD1 and SMA, focally positive for myogenin, while negative for h-caldesmon, SOX10 and S100P. A diagnosis of inflammatory leiomyosarcoma/HRRT was offered. Subsequently, the tumor was tested for MYOD1 (L122R) mutation and was found to be negative. The patient underwent adjuvant radiation therapy and is free-of-disease at 12 months post-treatment.

This case constitutes an extremely rare case of an inflammatory LMS/HRRT, identified in the neck region. This tumor should be differentiated from its close mimics, such as a spindle cell/sclerosing rhabdomyosarcoma, as the latter is treated more aggressively, including with chemotherapy, given its relatively poor prognosis.

## INTRODUCTION

An inflammatory leiomyosarcoma (LMS) is an extremely uncommon malignant mesenchymal tumor, presently included within the tumors of smooth muscle lineage (1). This is mostly reported in adult males, in sites such as the deep soft tissues of the lower limb, trunk, proximal limbs and in the retroperitoneum, followed by rare sites such as lung, ovary and parapharyngeal region, the latter sites in the form of isolated cases (1–5).

Histopathologically, inflammatory LMS is characterized by myogenic differentiation, accompanied by a prominent inflammatory component and genetically displays near-haploidization (1,2). Recent studies have shown a variable amount of rhabdomyoblastic differentiation within this tumor (3,5–7). Recently, Cloutier et al. (7) have shown “kinship” between an inflammatory LMS and a histiocyte-rich rhabdomyoblastic tumor (HRRT).

To the best of our knowledge, only two cases of HRRT have been reported in the neck region of adult males (7).

## CASE PRESENTATION

A 17-year-old male presented with complaints of a soft tissue lump over the back of his neck of one-year duration, which seemed to be increasing in size over the last 6 months.

**Figure 1:**
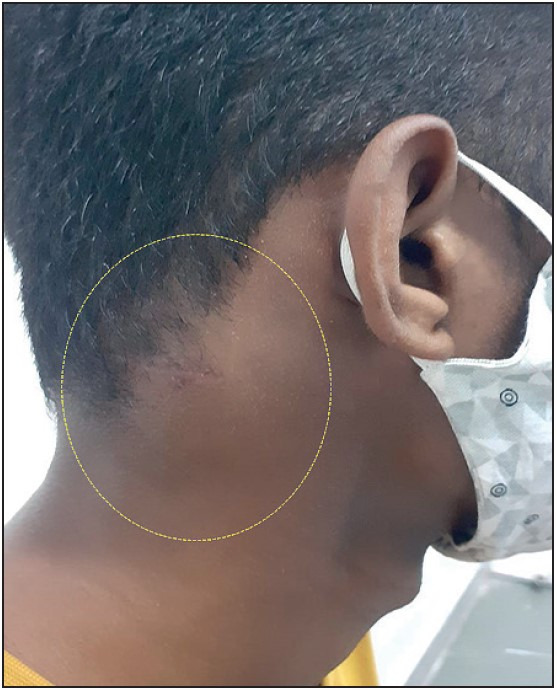
Clinical photograph showing a lump in the right side of neck behind the mastoid region, along with a healed horizontal scar of the previous biopsy.

On clinical examination, a firm, immobile lump over the right side of his neck behind the mastoid area was noted, measuring 5 cm x 4 cm. In addition, a healed horizontal scar of the previous open biopsy was noted. There was no neurological deficit. There was no other lesion elsewhere in his body (Figure 1).

**Figure 2:**
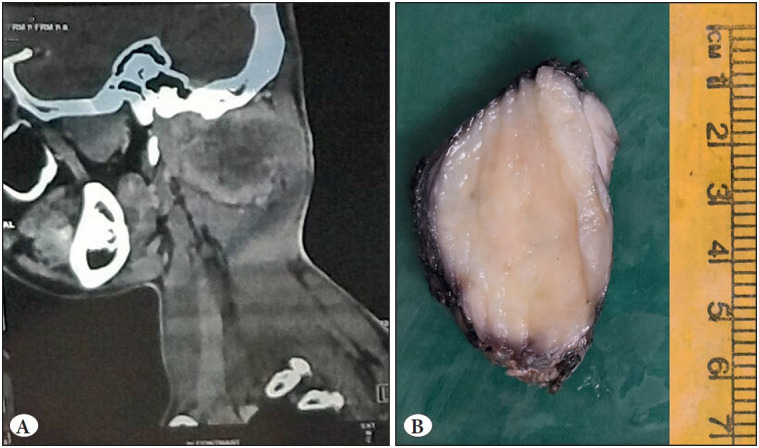
**A)** MRI revealing a lesion measuring 5.9 cm in the largest dimension, extending from the skull base superiorly up to the C2 vertebral level, abutting the occipital bone**. B)** Cut surface of the resected tumor showing a wellcircumscribed border with a greyA B white homogenous surface.

Computed tomography (CT) scan revealed a well-defined, hypodense lesion in the intermuscular plane over the postero-inferior and lateral aspect of the occipital region, extending up to the right mastoid region. On magnetic resonance imaging (MRI), there was a lesion measuring 5.3 cm x 5.9 cm x 5.0 cm, extending from the skull base superiorly up to the C2 vertebral level, abutting the occipital bone, leading to its thinning. Medially, the lesion was seen extending into the interlaminar space and neural foramina of C1-C2 vertebra, and above the C1 lamina. It is seen abutting the thecal sac. The lesion abutted the V3 or extraspinal segment of the right vertebral artery (~ 180 degrees). Anteriorly, it was seen extending up to the carotid space. There was no intracranial extension and/or spinal cord compression (Figure 2).

An incisional biopsy, performed and then reported as a fibrohistiocytic neoplasm at the referring laboratory, was reviewed at our Institution. Thereafter, the patient underwent an excision.

### Gross Findings

An unoriented specimen measuring 7.5 cm x 6.5 cm x 5 cm was received. On serial sectioning, a tumor was identified measuring 7.5 cm in the largest dimension. The cut surface was homogeneous, whitish in appearance and firm in consistency. There were no areas of necrosis and hemorrhage (Figure 2).

### Microscopic Findings

**Figure 3:**
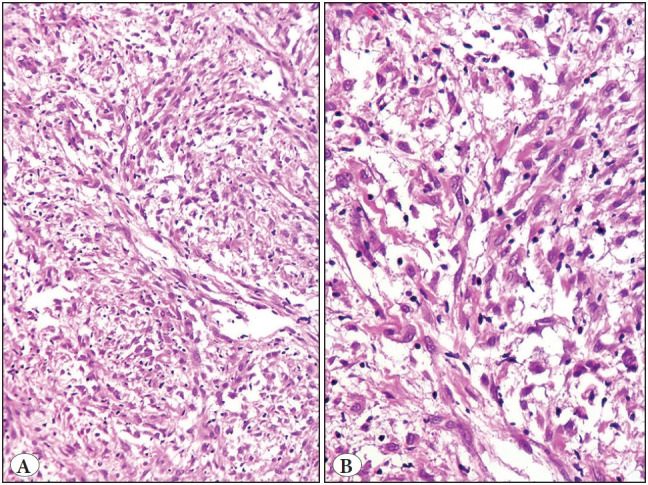
Microscopic findings (Biopsy). **A)** Cellular tumor composed of intersecting fascicles of spindle cells with intervening inflammatory cells. (H&E, x 200). **B)** Higher magnification showing plump spindle cells with tapering eosinophilic cytoplasm and scattered lymphocytes and histiocytes. (H&E, x 400).

**Figure 4:**
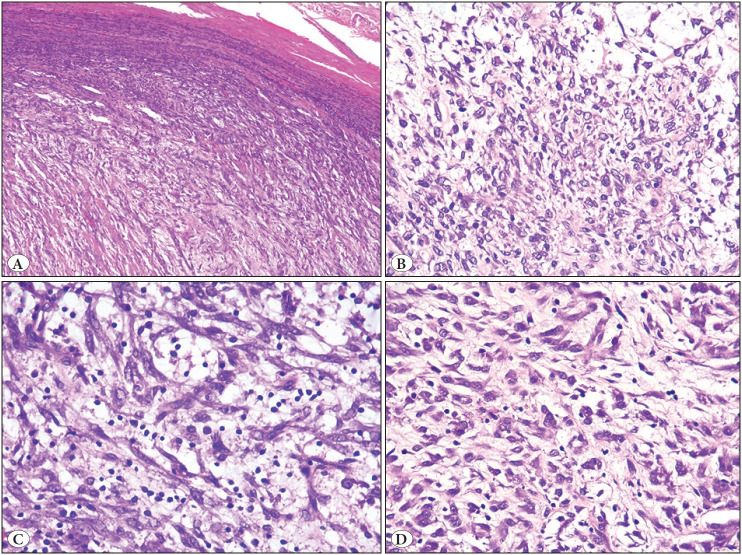
Microscopic findings (resected specimen). **A)** A circumscribed tumor with a well-defined pseudocapsule. (H&E, x 100). **B)** A cellular tumor displaying cells with spindle and polygonal shapes, and interspersed histiocytes and lymphocytes. (H&E, x 400). **C)** Foamy histiocytes and lymphocytes admixed with spindle-shaped tumor cells. (H&E, x 400). **D)** Tumor cells containing moderate to abundant cytoplasm with tapering ends, vesicular nuclei, exhibiting minimal nuclear atypia and interspersed histiocytes. (H&E, x 400).

Tissue sections revealed a well-circumscribed cellular tumor, with a thin pseudocapsule, composed of plump, spindle-shaped and polygonal or epithelioid cells, arranged in fascicles and bundles, and occasionally separated by a variable amount of hyaline and focally myxoid stroma. The individual tumor cells displayed oval to elongated nuclei, distinct nucleoli and moderate to abundant amount of eosinophilic to amphophilic cytoplasm with tapering ends. There was lack of significant nuclear atypia, mitotic figures, tumor necrosis and cells with cross striations on extensive tissue sampling. In addition, several histiocytes, including foamy cells and lymphocytes were interspersed throughout the tumor, obscuring the tumor cells in certain areas. Focal areas of tumor cells infiltrating the skeletal muscles were noted (Figure 3, Figure 4).

**Figure 5:**
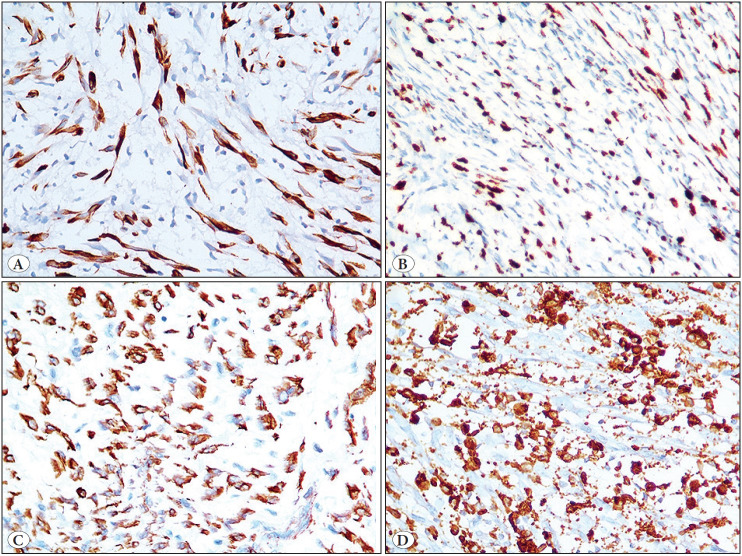
Immunohistochemical results. **A)** Tumor cells displaying desmin positivity. Diaminobenzidine, x 400. **B)** Multifocal MYOD1 positivity. (DAB, x 400). **C)** SMA positivity. DAB, x 400. **D)** CD163 highlighting several interspersed histiocytes and few tumor cells. (DAB, x 400).

**Figure 6:**
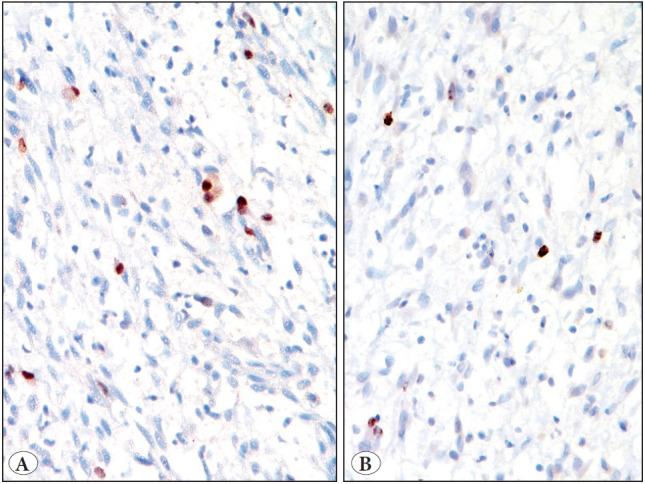
Immunohistochemical results. Focal myogenin positivity. (DAB, x 200). **B)** Low Ki67/MIB1. (DAB, x 400)

**Figure 7:**
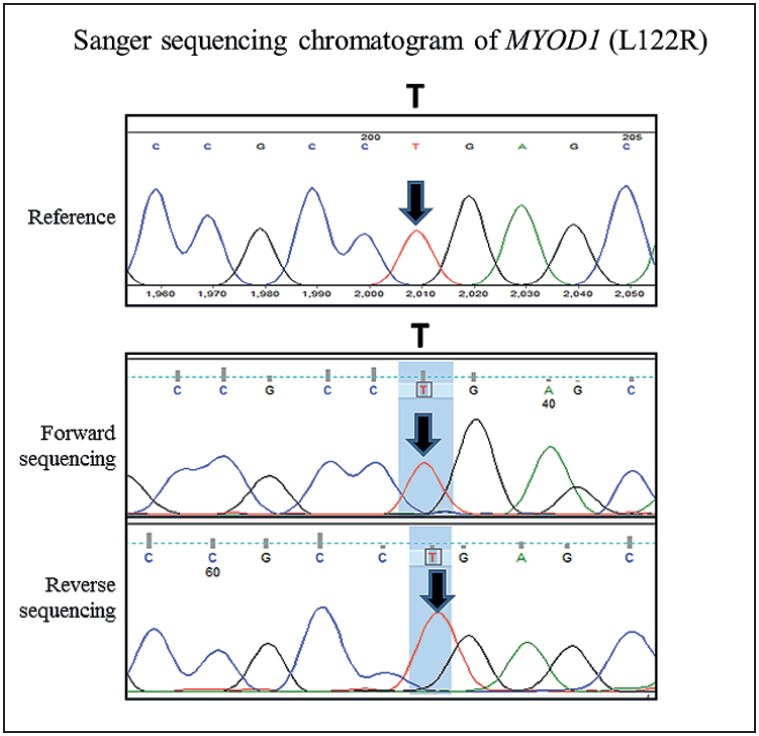
Sanger sequencing chromatogram of MYOD1 shown with reference sequence. No mutation found with forward and reverse sequencing reads. Arrow indicates nucleotide position.

Immunohistochemically, the tumor cells were positive for desmin (monoclonal, D33), MYOD1 (multifocal staining) (monoclonal, 5.8A); focally expressed smooth muscle actin (SMA) (monoclonal, 1A4) and myogenin (monoclonal, L026), while negative for S100 protein, SOX10, heavy isoform of caldesmon (H caldesmon) (monoclonal, h-CD) and ALK (monoclonal, D5F3). SMARCB1/INI1 expression was retained. Additionally, CD68 and CD163 highlighted numerous interspersed histiocytes (Figure 5, Figure 6). A diagnosis of an inflammatory LMS/HRRM was offered on the biopsy and further confirmed on the resection. In addition, the tumor was tested for *MYOD1 (L122R)* mutation by polymerase chain reaction (PCR), using forward and backward primers (8), followed by Sanger sequencing and was proved to be negative for *MYOD1 (L122R)* mutation (Figure 7).

The tumor was focally abutting the cauterized surface. Therefore, the patient was offered adjuvant radiation therapy. He has been free of disease for the last 12 months.

## DISCUSSION

Inflammatory LMS is a recently included neoplasm in the category of smooth muscle tumors in the current WHO classification of tumors of the soft tissues. At the same time, this tumor entity has created an interesting discussion, regarding its terminology, in view of its histogenesis and its clinical behavior. In two different studies, the authors have challenged its smooth muscle lineage and proposed alternate terminologies, such as “low-grade myofibroblastic tumor” and more recently, “inflammatory rhabdomyoblastic tumor”, including proximity between an inflammatory LMS and a histiocyte-rich rhabdomyoblastic tumor (HRRT), given that these tumors exhibit striking skeletal muscle differentiation (5,6). The present case constitutes the first case of an inflammatory leiomyosarcoma/HRRT, identified in the neck of an adolescent male, who was initially diagnosed with a fibrohistiocytic tumor at the referring laboratory. Previously, only two cases of HRRT have been reported, in a 45-year-old male and 26-year-old male (6).

Histopathologically, the presence of plump spindled cells in the present case led to various differential diagnoses, such as an inflammatory myofibroblastic tumor (IMFT), malignant peripheral nerve sheath tumor (MPNST) and a spindle cell/sclerosing rhabdomyosarcoma. Although there were plump cells with an abundant eosinophilic cytoplasm and tapering ends, resembling rhabdomyoblasts, there were no cross striations identified. Immunohistochemically, apart from desmin and focal SMA immunoreactivity, tumor cells also displayed multifocal myoD1 and focal myogenin positivity. This ruled out an IMFT. Moreover, the tumor cells were negative for ALK overexpression. Lack of S100P and SOX10 positivity ruled out an MPNST. However, spindle cell/sclerosing RMS was a close differential diagnosis, given the location and distinct rhabdomyoblastic differentiation. Despite rhabdomyoblastic differentiation, the lack of significant nuclear atypia, mitotic figures and a conspicuous amount of inflammatory component, including histiocytes, throughout the tumor, raised the possibility of spindle cell/ sclerosing RMS, although less likely. The inflammatory component was composed of mature lymphocytes and macrophages. In one of the earlier series, the authors observed that the lymphocytes were mostly CD3-positive T lymphocytes, along with a small population of CD20-positive B lymphocytes. Furthermore, Ki-67/MIB1 highlighted 1-2% of tumor cell nuclei (low). Similarly, Michal et al. (5) observed low Ki67/MIB1 in all five tumors in their study. Moreover, MYOD1 immunostaining revealed multifocal positivity in the present case, in contrast to diffuse staining that is reported in most spindle cell/sclerosing rhabdomyosarcomas (8–12). Presence of an admixture of plump spindle cells and inflammatory cells had led to an erroneous diagnosis of granulomatous inflammation on FNAC at the referring laboratory. Much earlier, these tumors were misdiagnosed as inflammatory malignant fibrous histiocytomas (1). The other morphological features described in an inflammatory LMS are Touton type of giant cells and focal psammomatous calcification (2,5,7).

Furthermore, we tested the present case for the *MYOD1 (LI22R)* mutation that constitutes a characteristic mutation underlying most cases of spindle cell/sclerosing RMS (8,12). The absence of *MYOD1 (L122R)* mutation was additionally useful in ruling out a spindle cell RMS, as similarly reported in five cases of HRRT, earlier by Martinez et al. (6).

During the initial description of an inflammatory LMS, Merchant et al. (1) observed a consistent immunoreactivity in the tumor cells for desmin, and variable immunopositivity for SMA and HHF-35. Chang et al. (4) reported negative immunoexpression for myogenin in all of their three study cases. Subsequently, using global gene expression profiling, Arbajian et al. (3) demonstrated a conspicuous differential expression of genes involved in muscle differentiation and function, including those of skeletal muscle differentiation, namely *ITGA7*, *MYF5*, *MYF6*, *MYO1*, *MYOG* and *PAX7 *in an inflammatory LMS. Thereafter, Michal et al. (5) reported positive immunostaining for MYOD1, myogenin and PAX7 in nine cases of inflammatory LMS. In view of co-expression of smooth and skeletal muscle specific markers, they proposed a reclassification of this tumor as a low-grade inflammatory myogenic tumor. Cloutier et al. (7) demonstrated proximity between an inflammatory LMS and histiocyte-rich rhabdomyoblastic tumor (HRRT), in the form of co-expression of desmin, SMA, MYOD1 and myogenin in four cases of inflammatory LMS and nine cases of HRRT and proposed reclassifying these tumors as inflammatory rhabdomyoblastic tumors. Apart from desmin, myogenin and MYOD1 positivity, none of the tumors in their study was positively immunoreactive for h caldesmon, which is considered as the most specific immunohistochemical marker of smooth muscle differentiation, as similarly noted in the present case (4,6,7,13). Chang et al. (4)( suggested that inflammatory leiomyosarcomas might lack smooth muscle differentiation. On the other hand, Arbajian et al. (3) and Michal et al. (5), reported h-caldesmon positivity in 3/4 cases and 5/8 cases of inflammatory LMS, in two different studies, respectively. However, there was a difference in the clone of h-Caldesmon in those studies (3,5). Similar to the study by Cloutier et al. (7), we tested the current tumor with the h-CD clone of h-caldesmon, rather than E89.

Apart from the positive immunoexpression of skeletal muscle specific markers, these tumors display significant number of CD68 and or CD163 positive histiocytes that seem to obscure the tumor cells, as well as expressed by some tumor cells, as observed in the present case and also in the previously reported cases. (1,3,5–7). It would be worth exploring whether the inflammatory component, including lymphocytes and macrophages are reactive or tumor-associated cells. This might have a possible bearing on the outcome of these tumors.

The importance of identifying this rare tumor is in view of its relatively favorable prognosis, unlike a spindle cell/sclerosing RMS that invariably shows an aggressive clinical course, in adult patients, especially those displaying the *MYOD1* (L122R) mutation (3,7,8,12). Post-excision and adjuvant radiation therapy, the present case has been free of disease for a year. None of the five cases harboring tumors in the somatic soft tissues in the study by Michal et al. (5) developed recurrences or metastasis. Similarly, none of the nine previously reported cases in three different studies developed tumor recurrences or metastasis over a period of 5-120 months (3,6,7).

Regarding its genetic profiling, Dal Cin et al. (14) reported near-haploid genotype in two cases of inflammatory LMS. Subsequently, various authors demonstrated similar results in various cases of inflammatory LMS (3,4,7,15). Despite most chromosomes showing loss of heterozygosity, heterozygosity for chromosomes 5 and 22 and frequently for 18, 20 and 21 chromosomes has been reported to be retained in this tumor (3,4,6,14,15). Moreover, oncogenic inactivating mutations in the NF1 gene have been reported in few cases of inflammatory LMS and HRRTs (5,6). In addition, Martinez et al. (6) reported a likely benign *PTCH1* polymorphism (rs115556836:c.2183C>T:pThr728Met) in a single case of HRRT. Unfortunately, genetic profiling was not performed in the recent case.

**In conclusion**, the present case constitutes a rare case of an inflammatory leiomyosarcoma/HRRT, identified in the neck of an adolescent male. Given the diagnosis of an inflammatory LMS, the patient was spared of intensive chemotherapy that is invariably offered to most cases of RMS, especially large-sized tumors. Although extremely rare, this entity should be included in the differential diagnosis of a spindle cell tumor showing co-expression of smooth and skeletal muscle markers, with a prominent histiocytic component and rare mitoses. An exact diagnosis of this tumor with an evolving terminology has significant treatment-related implications.

## Conflict of Interest

We declare that we have no conflict of interest.

## Authorship Contributions

Concept, design, data collection, analysis/interpretation, literature search, writing and approval: **BR,** Data collection, analysis, writing and approval: **MB,** Interpretation, writing and approval: **BD,** Interpretation writing and approval: **AD,** Data collection, writing and approval: **PP.**

## References

[ref-1] Merchant W., Calonje E., Fletcher C. D. (1995). Inflammatory leiomyosarcoma: a morphological subgroup within the heterogeneous family of so-called inflammatory malignant fibrous histiocytoma. Histopathology.

[ref-2] Fletcher CDM, Mertens F, Editorial Board World Health Organization (WHO) Classification of Tumours (2020). Inflammtory leiomyosarcoma. World Health Organization classification of tumours.

[ref-3] Arbajian Elsa, Köster Jan, Steyern Fredrik, Mertens Fredrik (2018). Inflammatory leiomyosarcoma is a distinct tumor characterized by near-haploidization, few somatic mutations, and a primitive myogenic gene expression signature. Mod Pathol.

[ref-4] Chang Anthony, Schuetze Scott M., Conrad Ernest U., Swisshelm Karen L., Norwood Thomas H., Rubin Brian P. (2005). So-called "inflammatory leiomyosarcoma'': a series of 3 cases providing additional insights into a rare entity. Int J Surg Pathol.

[ref-5] Michal Michael, Rubin Brian P., Kazakov Dmitry V., Michalová Květoslava, Šteiner Petr, Grossmann Petr, Hájková Veronika, Martínek Petr, Švajdler Marian, Agaimy Abbas, Hadravský Ladislav, Kalmykova Antonina V., Konishi Eiichi, Heidenreich Filip, Michal Michal (2020). Inflammatory leiomyosarcoma shows frequent co-expression of smooth and skeletal muscle markers supporting a primitive myogenic phenotype: a report of 9 cases with a proposal for reclassification as low-grade inflammatory myogenic tumor. Virchows Arch.

[ref-6] Martinez Anthony P., Fritchie Karen J., Weiss Sharon W., Agaimy Abbas, Haller Florian, Huang Hsuan-Ying, Lee Seungjae, Bahrami Armita, Folpe Andrew L. (2019). Histiocyte-rich rhabdomyoblastic tumor: rhabdomyosarcoma, rhabdomyoma, or rhabdomyoblastic tumor of uncertain malignant potential? A histologically distinctive rhabdomyoblastic tumor in search of a place in the classification of skeletal muscle neoplasms. Mod Pathol.

[ref-7] Cloutier Jeffrey M., Charville Gregory W., Mertens Fredrik, Sukov William, Fritchie Karen, Perry Kyle D., Edgar Mark, Rowsey Ross A., Folpe Andrew L. (2021). "Inflammatory Leiomyosarcoma" and "Histiocyte-rich Rhabdomyoblastic Tumor": a clinicopathological, immunohistochemical and genetic study of 13 cases, with a proposal for reclassification as "Inflammatory Rhabdomyoblastic Tumor". Mod Pathol.

[ref-8] Rekhi Bharat, Upadhyay Pawan, Ramteke Manoj P., Dutt Amit (2016). MYOD1 (L122R) mutations are associated with spindle cell and sclerosing rhabdomyosarcomas with aggressive clinical outcomes. Mod Pathol.

[ref-9] Rubin B. P., Hasserjian R. P., Singer S., Janecka I., Fletcher J. A., Fletcher C. D. (1998). Spindle cell rhabdomyosarcoma (so-called) in adults: report of two cases with emphasis on differential diagnosis. Am J Surg Pathol.

[ref-10] Nascimento Alessandra F., Fletcher Christopher D. M. (2005). Spindle cell rhabdomyosarcoma in adults. Am J Surg Pathol.

[ref-11] Rekhi Bharat, Singhvi Tanvi (2014). Histopathological, immunohistochemical and molecular cytogenetic analysis of 21 spindle cell/sclerosing rhabdomyosarcomas. APMIS.

[ref-12] Szuhai Karoly, Jong Daniëlle, Leung Wai Yi, Fletcher Christopher D. M., Hogendoorn Pancras C. W. (2014). Transactivating mutation of the MYOD1 gene is a frequent event in adult spindle cell rhabdomyosarcoma. J Pathol.

[ref-13] Watanabe K., Tajino T., Sekiguchi M., Suzuki T. (2000). h-Caldesmon as a specific marker for smooth muscle tumors. Comparison with other smooth muscle markers in bone tumors. Am J Clin Pathol.

[ref-14] Dal Cin P., Sciot R., Fletcher C. D., Samson I., De Vos R., Mandahl N., Willén H., Larsson O., Berghe H. (1998). Inflammatory leiomyosarcoma may be characterized by specific near-haploid chromosome changes. J Pathol.

[ref-15] Nord Karolin H., Paulsson Kajsa, Veerla Srinivas, Wejde Johan, Brosjö Otte, Mandahl Nils, Mertens Fredrik (2012). Retained heterodisomy is associated with high gene expression in hyperhaploid inflammatory leiomyosarcoma. Neoplasia.

